# Supplementary material for: Tryptophan and Kynurenine Enhances the Stemness and Osteogenic Differentiation of Bone Marrow-Derived Mesenchymal Stromal Cells In Vitro and In Vivo
Source: Materials (Basel). 2021 Jan 4;14(1):208. doi: 10.3390/ma14010208 (PMC7796421; doi:10.3390/ma14010208)
Supplement: Supplementary file 1 [file materials-14-00208-s001.pdf]

materials

Supplementary Material

Tryptophan and Kynurenine Enhances the Stemness and Osteogenic Differentiation of Bone Marrow-Derived Mesenchymal Stromal Cells In Vitro and In Vivo

Hai Thanh Pham [1,4], Mitsuaki Ono [2,*], Emilio Satoshi Hara [3,*], Ha Thi Thu Nguyen [1,2,4], Anh Tuan Dang [1,2,4], Hang Thuy Do [1,2,4], Taishi Komori [1], Ikue Tosa [1], Yuri Hazehara-Kunitomo [1,2], Yuya Yoshioka [1], Yasutaka Oida [1], Kentaro Akiyama [1] and Takuo Kuboki [1]

[1] Department of Oral Rehabilitation and Regenerative Medicine, Dentistry and Pharmaceutical Sciences, Okayama University Graduate School of Medicine, Okayama 700-8558, Japan; pth_uytki@yahoo.com (H.T.P.); thuharhm@gmail.com (H.T.T.N.); ppjj5xuj@s.okayama-u.ac.jp (A.T.D.); pa62884x@s.okayama-u.ac.jp (H.T.D.); de19016@s.okayama-u.ac.jp (T.K.); de421035@s.okayama-u.ac.jp (I.T.); de19034@s.okayama-u.ac.jp (Y.H.-K.); de17054@s.okayama-u.ac.jp (Y.Y.); gmd20103@s.okayama-u.ac.jp (Y.O.); akentaro@md.okayama-u.ac.jp (K.A.); kuboki@md.okayama-u.ac.jp (T.K.)

[2] Department of Molecular Biology and Biochemistry, Dentistry and Pharmaceutical Sciences, Okayama University Graduate School of Medicine, Okayama 700-8558, Japan

[3] Department of Biomaterials, Dentistry and Pharmaceutical Sciences, Okayama University Graduate School of Medicine, Okayama 700-8558, Japan

[4] Faculty of Dentistry, Hai Phong University of Medical and Pharmacy, Haiphong 04211, Vietnam

* Correspondence: mitsuaki@md.okayama-u.ac.jp (M.O.); gmd421209@s.okayama-u.ac.jp (E.S.H.); Tel.: +81-86-235-7127 (M.O.); +81-86-235-6667 (E.S.H.); Fax: +81-86-222-7768 (M.O.); +81-86-235-6669 (E.S.H.)

* Correspondence: mitsuaki@md.okayama-u.ac.jp (M.O.); gmd421209@s.okayama-u.ac.jp (E.S.H.); Tel.: +81-86-235-7128 (M.O.); Tel.: +81-86-235-6667 (E.S.H.); Fax: +81-86-222-7768 (M.O.); Fax: +81-86-235-6669 (E.S.H.)

Citation: Pham, H.T.; Ono, M.; Hara, E.S.; Nguyen, H.T.T.; Dang, A.T.; Do, H.T.; Komori, T.; Tosa, I.; Hazehara-Kunitomo, Y.; Yoshioka, Y.; et al. Tryptophan and Kynurenine enhances the stemness and osteogenic differentiation of bone marrow-derived mesenchymal stromal cells in vitro and in vivo. *Materials* **2020**, *14*, x. https://doi.org/10.3390/xxxxx

Received: 1 December 2020
Accepted: 21 December 2020
Published: date

Publisher's Note: MDPI stays neutral with regard to jurisdictional claims in published maps and institutional affiliations.

Copyright: © 2020 by the authors. Licensee MDPI, Basel, Switzerland. This article is an open access article distributed under the terms and conditions of the Creative Commons Attribution (CC BY) license (http://creativecommons.org/licenses/by/4.0/).

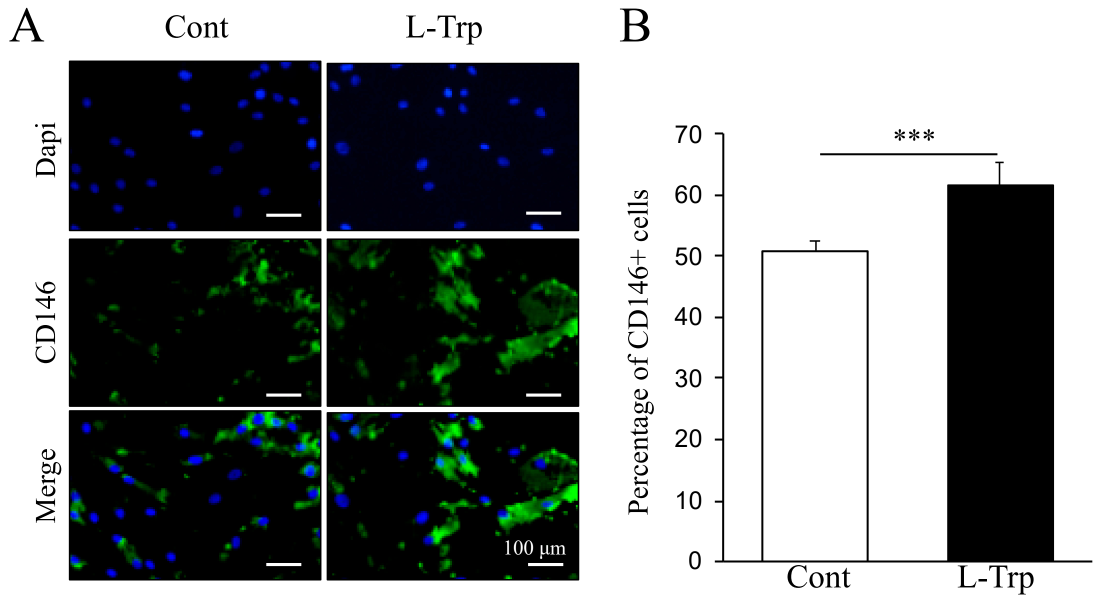

Figure S1. Effect of L-Tryptophan on the stem cell phenotype of hBMSCs. Cells were treated with L-Trp for two days, and then the expression of CD146 was analyzed in the cells. A higher number of $CD146^{+}$ cells was observed in the L-Trp-treated hBMSCs, as shown by the fluorescence images **(A)** and quantitative analysis **(B)**. DAPI = blue. CD146 = green. Data represent the mean ± SD (n = 3). *** $p < 0.001$, unpaired *t*-test.

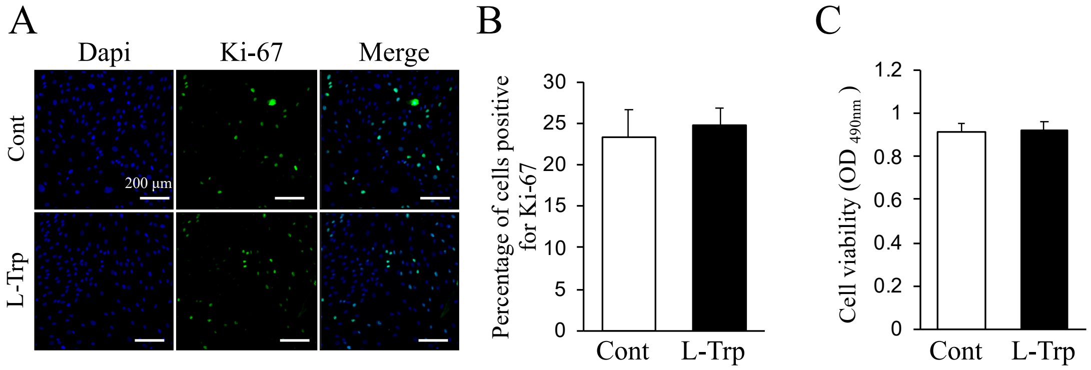

Figure S2. Effect of L-Tryptophan on cell cycle and cell viability. (**A**) No change in the expression of the cell proliferation marker Ki-67 was detected after the treatment of L-Trp. DAPI and Ki-67 are shown in green and blue, respectively. Images and graph are representatives of at least 3 independent experiments. **(B)** L-Trp did not affect cell viability performed by MTS assay. Graph is representative of at least 3 independent experiments.

Table S1. List of D-isomer amino acids included in the orphan ligand library that was used in the screening.

ORPHAN LIGANDS					
**#**	**CAS #**	**Name**	**M.W.**	**Conc. (mMOL)**	**Rationale**
1	1783-96-6	D-Aspartic acid	133.1	10	Putative endogenous ligand
2	6893-26-1	D-Glutamic acid	147.1	10	Putative endogenous ligand
3	312-84-5	D-Serine	105.1	10	Putative endogenous ligand
4	338-69-2	D-Alanine	89.1	10	D-Amino acid
5	6729-55-1	g-D-Glutamylglycine	204.2	10	Putative endogenous ligand
6	157-06-2	D-Arginine	174.2	10	D-Amino acid
7	5794-24-1	D-Asparagine	150.1	10	D-Amino acid

8	921-01-7	D-Cysteine	121.1	10	D-Amino acid
9	5959-95-5	D-Glutamine	146.1	10	D-Amino acid
10	351-50-8	D-Histidine	155.2	10	D-Amino acid
11	319-78-8	D-Isoleucine	131.2	10	D-Amino acid
12	328-38-1	D-Leucine	131.2	10	D-Amino acid
13	923-27-3	D-Lysine	146.2	10	D-Amino acid
14	348-67-4	D-Methionine	149.2	10	D-Amino acid
15	16682-12-5	D-Ornithine	168.6	10	D-Amino acid
16	673-06-3	D-Phenylalanine	165.2	10	D-Amino acid
17	344-25-2	D-Proline	115.1	10	D-Amino acid
18	4042-36-8	D-Pyroglutamic acid	129.1	10	D-Amino acid
19	632-20-2	D-Threonine	119.1	10	D-Amino acid
20	153-94-6	D-Tryptophan	204.2	10	D-Amino acid
21	556-02-5	D-Tyrosine	181.2	10	D-Amino acid
22	640-68-6	D-Valine	117.1	10	D-Amino acid

Table S2. List of primer sequences used for quantitative analysis of gene expression levels by real-time RT-PCR.

Gene	Species	Gene Bank Accession no.	Primer Sequence	PCR Product Length (bp)
S29	Human	BC032813	5'-TCTCGCTCTTGTCGTGTCTGTTC-3'(S)<br>5'-ACACTGGCGGCACATATTGAGG-3'(AS)	75
OCT-4	Human	NM_001159542	5'-CCGAGTGTGGTTCTGTAAC-3'(S)<br>5'-GAAAGGGACCGAGGAGTA-3'(AS)	196
NANOG	Human	NM_024865	5'-TCTCCAACATCCTGAACCT-3'(S)<br>5'-GCGTCACACCATTGCTAT-3'(AS)	117
SOX-2	Human	NM_003106	5'-GCACAACTCGGAGATCAG-3'(S)<br>5'-GCGTGTACTTATCCTTCTTCAT-3'(AS)	182
OPN	Human	BC007016	5'-CTGTGTTGGTGGAGGATGTCTGC-3'(S)<br>5'-GTCGGCGTTTGGCTGAGAAGG-3'(AS)	89
OCN	Human	NM_199173	5'-CAGAGTCCAGCAAAGGTG-3'(S)<br>5'-AGCCATTGATACAGGTAGC-3'(AS)	88
LPL	Human	BT006726	5'-CCGCCGCCGACCAAAGAAG-3'(S)<br>5'-GAAATGACAGGTAGCCACGGACTC-3'(AS)	131
PPARγ	Human	BT007281	5'-CTGTCGGTTTCAGAAATGCCTTGC-3'(S)<br>5'-GGAGGTCAGCGGACTCTGGATTC-3'(AS)	143
Oct-4	Mouse	NM_013633	5'-GCATTGAGAACCGTGTGA-3' (S)<br>5'-GATTGGCGATGTGAGTGAT-3'(AS)	81
Sox2	Mouse	NM_011443	5'-CTCGCAGACCTACATGAAC-3'(S)<br>5'-CTCGGACTTGACCACAGA-3(AS)'	106
Nanog	Mouse	NM_028016	5'-TTGGTGTGTTAGTGTATTTGTC-3'(S)<br>5'-TGGGAAGGAGAGAGTATATGC-3'(AS)	101
Opn	Mouse	AF515708	5'-GAATGCTGTGTCCTCTGAAG-3'(S)<br>5'-CGTCATCATCATCGTCATCAT-3'(AS)	110
Ocn	Mouse	BC055868	5'-CCAAGCAGGAGGGCAATAAGGTAG-3'(S)<br>5'-CTCGTCACAAGCAGGGTCAAGC-3' (AS)	122
Pparγ	Mouse	NM_01116	5'-GCCAAGGTGCTCCAGAAGATGAC-3'(S)	155

			5'-CGGGTGGGACTTTCCTGCTAATAC-3'(AS)	
Lpl	Mouse	BC003305	5'-CTCTTTATTGACTCTCTGCTGAA-3'(S)	110
			5'-GGCTCTGACCTTATTGATCTC-3'(AS)	
S29	Mouse	NM_009093	5'-TTCCTTTCTCCTCGTTGG-3'(S)	108
			5'-ATGTTCAGCCCGTATTTG-3'(AS)	
**S: sense; AS: antisense**				
